# Secreted miR-210-3p, miR-183-5p and miR-96-5p reduce sensitivity to docetaxel in prostate cancer cells

**DOI:** 10.1038/s41420-023-01696-4

**Published:** 2023-12-08

**Authors:** Maristella Canovai, Monica Evangelista, Alberto Mercatanti, Romina D’Aurizio, Letizia Pitto, Francesca Marrocolo, Valentina Casieri, Marco Pellegrini, Vincenzo Lionetti, Sergio Bracarda, Milena Rizzo

**Affiliations:** 1 Institute of Clinical Physiology (IFC), CNR, Pisa, Italy; 2 Institute for Informatics and Telematics (IIT), CNR, Pisa, Italy; 3 UOC Medical Oncology, San Donato Hospital, Arezzo, Italy; 4 Unit of Translational Critical Care Medicine, Laboratory of Basic and Applied Medical Sciences, Interdisciplinary Research Center “Health Science”, Sant’Anna school of Advanced Studies, Pisa, Italy; 5 Medical and Translational Oncology, Department of Oncology, Azienda Ospedaliera Santa Maria, Terni, Italy

**Keywords:** Prostate cancer, miRNAs, Cancer therapeutic resistance

## Abstract

Docetaxel (DCT) resistance is one of the main factors responsible for treatment failure in metastatic prostate cancer (PCa). Although several mechanisms of DCT resistance have been elucidated, the issue is still far from comprehensive. In this work we show that miR-96-5p, miR-183-5p and miR-210-3p (referred to as sDCT^R^-miRNAs) are specifically released by DCT resistant (DCT^R^) PCa clones and decrease the efficacy of DCT in PCa cells when overexpressed. Through bioinformatic analysis, we identified several potential targets of sDCT^R^-miRNAs’ activity including FOXO1, IGFBP3, and PDCD4 known to exert a role in DCT resistance. Additionally, we found that PPP2CB and INSIG1 mediated the ability of sDCT^R^-miRNAs to reduce the efficacy of DCT. We explored whether secreted sDCT^R^-miRNAs could affect the phenotype of PCa cells. We found that exposure to exosomes derived from DCT^R^ PCa clones (in which the content of sDCT^R^-miRNAs was higher than in exosomes from parental cells), as well as exposure to exosome loaded with sDCT^R^-miRNAs, reduced the cytotoxicity of DCT in PCa cells sensitive to the drug. Finally, we validated circulating miR-183-5p and miR-21-5p as potential predictive biomarkers of DCT resistance in PCa patients. Our study suggests a horizontal transfer mechanism mediated by exosomal miRNAs that contributes to reduce docetaxel sensitivity and highlights the relevance of cell-to-cell communication in drug resistance.

## Introduction

Recent data suggest an increased incidence of prostate cancer (PCa), which remains the second leading cause of cancer-related deaths in males in the United States [1]. While localized PCa has a very high 5-year survival rate, advanced PCa remains a largely incurable disease [2]. Typically, most advanced PCa patients are initially treated with androgen deprivation therapy; however, most of them develop resistance and progress to castration-resistant prostate cancer (CRPC) [3]. At this stage, despite the continued development of new therapies, docetaxel (DCT) remains the approved choice due to its efficacy in prolonging lifespan [2, 4, 5]. Unfortunately, the efficacy of DCT treatment is also weakened by the development of resistance and cross-resistance phenomena [4, 6].

Resistance to DCT is a multifactorial process that may depend on mechanisms related to the biology of PCa or on more general mechanisms of drug resistance that occur in tumors. Indeed, DCT resistance may be due to the following: increased drug efflux transporters such as MDR1 or other members of the ATP-binding cassette (ABC) transporter family; structural/functional alterations in microtubules; induction of pro-survival and/or apoptosis escape signaling pathways; alteration in androgen receptor signaling; increase in stem cell population; hypoxic signaling and strictly correlated activation of epithelial to mesenchymal transition (EMT) signaling [7–9]. In recent years, several reports have been published on the role of miRNAs in this phenomenon [10]. Most of these miRNAs affect DCT cytotoxicity by altering either DCT-induced apoptosis and pro-survival pathway or EMT signaling. Very recently, additional miRNAs involved in the above-mentioned DCT resistance mechanisms have been identified (e.g. [11–14]).

Although many mechanisms of DCT resistance have been elucidated, this topic is far from exhausted. Of particular interest is the role of tumor microenvironment (TME), in which a mixture of tumor and non-tumor cells coexist in a disorganized vascular network that influences drug uptake and induces environmental changes such as hypoxia that in turn promotes pro-survival pathways and reduces drug efficacy [15–17]. In this context, the crosstalk between the different cellular components of TME plays a pivotal role in the development of drug resistance, and exosomal miRNAs are involved as “signaling molecules” in this phenomenon [18] including DCT resistance [19, 20].

In this work, we showed the role of miR-96-5p, miR-183-5p, and miR-210-3p in reducing the efficacy of DCT in PCa cell lines. Since PCa cells resistant to DCT specifically release these miRNAs, their potential role in cell-to-cell communication and as biomarkers of DCT resistance in PCa patients was also investigated.

## Results

### Identification of miRNAs released by DCT resistant PCa clones

**Fig. 1:**
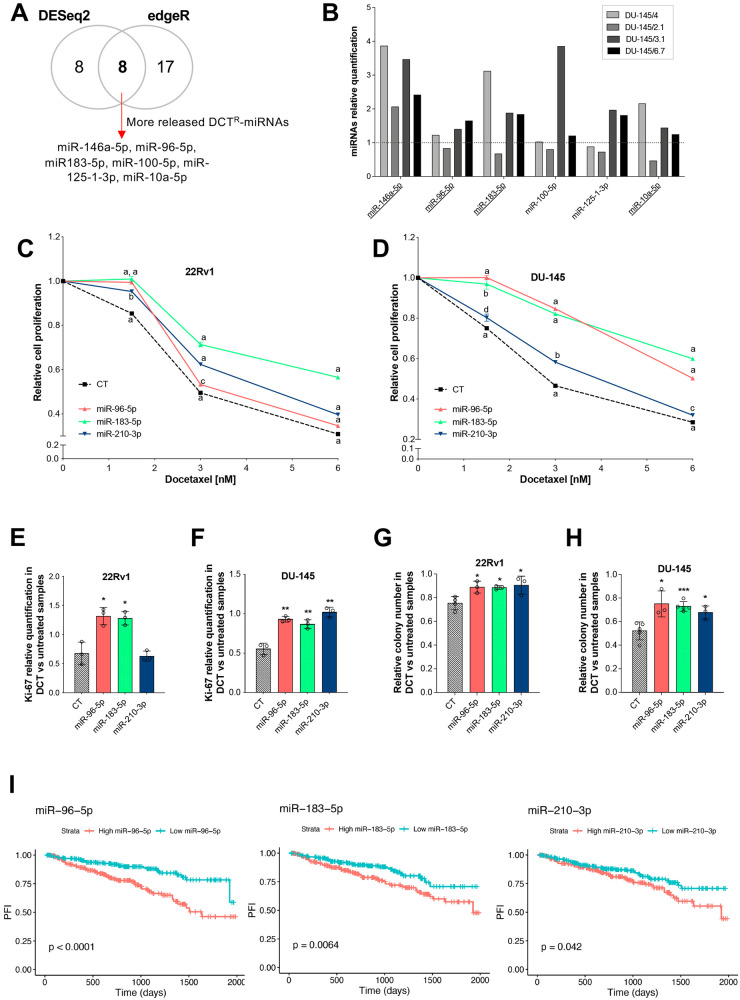
The sensitivity of PCa cells to DCT was affected by DCT^R^-miRNAs overexpression. **A** Schematic representation of the NGS data analysis used to identify the DCT^R^-miRNAs in DU-145/DCT^R^ clones. **B** DCT^R^-miRNAs relative quantification by qRT-PCR in the medium of DU-145/DCT^R^ clones compared to parental cells medium. Effect of DCT^R^-miRNAs overexpression on proliferation (**C**–**F**) and colony forming ability (**G**, **H**) in 22Rv1 (**C**, **E**, **G**) and DU-145 (**D**, **F**, **H**) cells. Cell proliferation (**C**, **D**) was evaluated for each miRNA in treated (with increasing DCT doses) compared to untreated samples. Ki-67 mRNA expression level (**E**, **F**) and colony forming ability (**G**, **H**) were evaluated for each miRNA in treated (3 nM DCT) compared to untreated sample. **I** Kaplan–Meier curves showing progression-free interval (PFI) relative to patients stratified by sDCT^R^-miRNAs expression level (high/low according to 0.5 quantiles of log2(total_RPM + 1)). Data are reported as mean ± SD of at least three independent experiments, *P < 0.05, **P < 0.01, ***P < 0.001, ****< 0.0001 unpaired *t*-test (**C**–**H**). In (**C**, **D**) ^d^*P* < 0.05, ^c^*P* < 0.01, ^b^*P* < 0.001, ^a^*P* < 0.0001 (unpaired) *t*-test.

Previously, we generated DCT resistant (DCT^R^) PCa clones from both an androgen-dependent (22Rv1) and -independent (DU-145) cell line [21]. Through miRNA profiling of cultured media of DU-145 and 22Rv1 DCT^R^ clones and their corresponding parental cell lines, we identified the miRNAs specifically released by all clones compared to parental cells (referred to as DCT^R^-miRNAs). We found that 22Rv1 DCT^R^ clones specifically released several miRNAs, while DU-145 DCT^R^ clones released only miR-146a-5p [21]. In this work, we decided to re-analyze the NGS data of DU-145 DCT^R^ clones. When considering the hierarchical cluster obtained from the normalized read counts of DU-145 DCT^R^ clones and parental cells (Fig. S1A), we observed that the miRNA profile of DCT^R^ clones 2A and 2B appeared to be more similar to DU-145 parental cells than to the other DU-145 DCT^R^ clones. Therefore, we excluded these two clones and identified the miRNAs that were released more abundantly only by DU-145 DCT^R^ clones 2.1, 3.1, 6.7, and 4. Using DESeq2 and edgeR methods we identified 16 (DESeq2, padj < 0.05) and 25 (edgeR, FDR < 0.05) miRNAs differentially released (Fig. 1A, Table S1). We considered only the miRNAs that were (i) more strongly released and (ii) identified by both methods, and we validated their levels by qRT-PCR (Fig. 1B). We selected the miRNAs more released from at least 3 out of 4 DCT^R^ clones (underlined in Fig. 1B). For completeness, we also applied the same differential analyses to DCT^R^ clones 2 A and 2B versus parental cells. As expected, DESeq2 and edgeR identified only miR-146a-5p as the common more released miRNA (Table S2). Table 1 shows the DCT^R^-miRNAs more released by (i) 22Rv1 (previously identified and validated) and (ii) DU-145 (according to the new analyses and validations) DCT^R^ clones.

**Table 1:** DCT^R^-miRNAs more released by PCa DCT^R^ clones.

22Rv1 DCT^R^ clones	DU-145 DCT^R^ clones
miR-210-3p	miR-146a-5p
miR-21-5p	miR-96-5p
miR-21-3p	miR-183-5p
	miR10a-5p

### DCT^R^-miRNAs modify the response of PCa cells to DCT

We hypothesized that DCT^R^-miRNAs could affect the response of PCa cells to DCT. We selected some DCT^R^-miRNAs (i.e. miR-210-3p, miR-96-5p and miR-183-5p, referred to as sDCT^R^-miRNAs) for further analyses, based on the biological processes in which they are involved. We overexpressed these miRNAs in both 22Rv1 and DU-145 cells (Fig. S1B) and examined cell proliferation at increasing DCT doses (Fig. 1C, D). The results suggested that all sDCT^R^-miRNAs reduce the chemosensitivity of PCa cells to DCT. Consistent with these findings, the expression of the proliferation marker Ki-67 was higher in sDCT^R^-miRNAs overexpressed cells (DCT treated versus untreated) than in CT transfected cells (Fig. 1E, F). We also evaluated the colony-forming ability of PCa cells overexpressing sDCT^R^-miRNAs after DCT treatment and found that sDCT^R^-miRNAs overexpression reduced the cytotoxic effect of DCT on colony formation (Fig. 1G, H). Overall, our results show that sDCT^R^-miRNAs protect PCa cells from DCT toxicity.

To evaluate the clinical relevance of the in vitro data, we analyzed the expression levels of sDCT^R^-miRNAs in the prostate adenocarcinoma dataset (PRAD) deposited in the Cancer Genome Atlas (TCGA). Interestingly, Kaplan–Meier analyses showed a significant difference in progression-free interval (PFI) (Fig. 1I) and disease-free interval (DFI) (Fig. S1D) between patient groups stratified by the expression level of sDCT^R^-miRNAs. In detail, patients with higher sDCT^R^-miRNAs had a significantly shorter free interval before the occurrence of disease progression or relapse.

### sDCT^R^-miRNAs decrease DCT sensitivity of PCa cells by regulating PPP2CB and INSIG

**Fig. 2:**
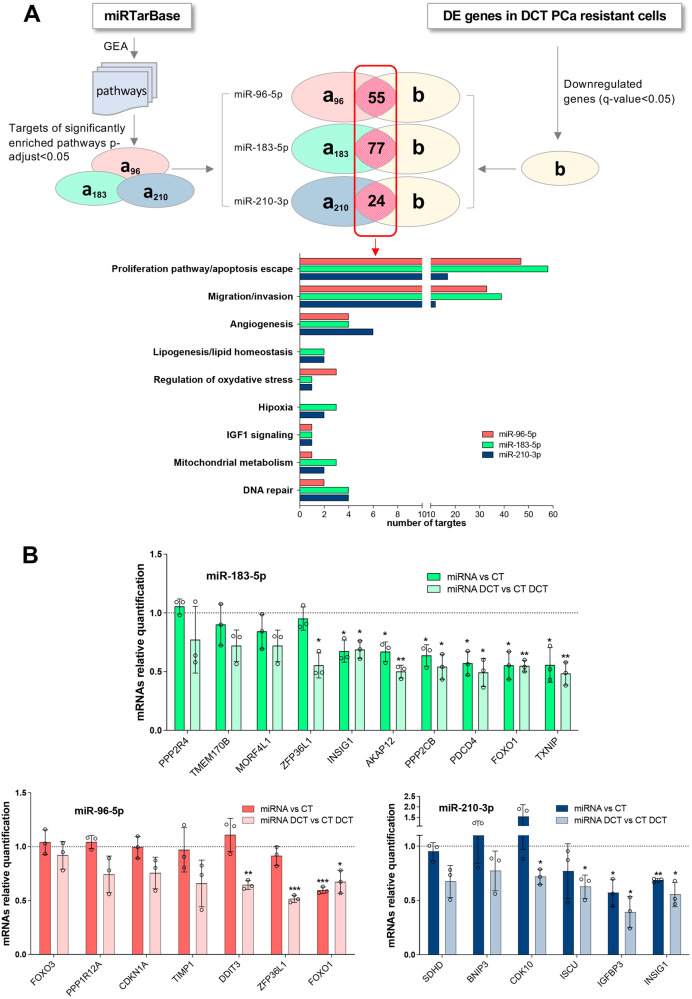
Molecular mediators of sDCT^R^-miRNAs effect on DCT response in PCa cells. **A** Schematic representation of the bioinformatic analysis to identify the putative mediator of sDCT^R^-miRNAs effect on DCT sensitivity. **B** Relative quantification by qRT-PCR of sDCT^R^-miRNA targets after sDCT^R^-miRNAs overexpression with or without 3 nM DCT. Data are reported as mean ± SD of at least three independent experiments, *P < 0.05, **P < 0.01, ***P < 0.001 unpaired *t*-test.

We investigated the possible molecular pathways through which sDCT^R^-miRNAs reduce DCT sensitivity. We considered the validated miRNA targets deposited in miRTarBase 8.0 and performed a gene enrichment analysis (GEA) using different databases (described in Materials and Methods). We selected the targets belonging to the pathways with *p*-adjust < 0.05 (Fig. 2A). To increase the probability of identifying the targets that play a role in DCT response, we exploited single-cell transcriptomic analyses performed on DCT resistant single cells derived from two PCa cell lines (PC-3 and DU-145) [22]. We looked at genes that were differentially expressed in DCT resistant cells (compared to sensitive cells) for each cell line. Because overexpression of sDCT^R^-miRNAs increases resistance to DCT and miRNAs are repressors of gene expression, we selected the sDCT^R^-miRNAs targets that were downregulated in both DU-145 and PC-3 DCT resistant cells (significant *q*-value < 0.05 in at least one cell type) (Fig. 2A, Table S3). Finally, we selected those targets whose downregulation was consistent with a decrease in DCT sensitivity (Table 2).

**Table 2:** List of selected sDCT^R^-miRNAs validated targets.

Targets	sDCT^R^-miRNA	Biological processes/pathways
FOXO3	miR-96-5p	Proliferation/apoptosis escape
CDKN1A	miR-96-5p	Proliferation/apoptosis escape
PPP1R12A	miR-96-5p	Proliferation/apoptosis escape
DDIT3	miR-96-5p	Proliferation/apoptosis escape
TIMP1	miR-96-5p	Migration/invasion
PDCD4	miR-183-5p	Proliferation/apoptosis escape
PPP2CB	miR-183-5p	Proliferation/apoptosis escape
PPP2R4	miR-183-5p	Proliferation/apoptosis escape
AKAP12	miR-183-5p	Proliferation/apoptosis escape; Migration/invasion
MORF4L1	miR-183-5p	Proliferation/apoptosis escape; Migration/invasion
TMEM170B	miR-183-5p	Proliferation/apoptosis escape; Migration/invasion
TXNIP	miR-183-5p	Proliferation/apoptosis escape; Regulation of oxidative stress
BNIP3	miR-210-3p	Proliferation/apoptosis escape
IGFBP3	miR-210-3p	Proliferation/apoptosis escape; Migration/invasion; IGF signaling
CDK10	miR-210-3p	Proliferation/apoptosis escape; Migration/invasion
SDHD	miR-210-3p	Mitochondrial metabolism; Hipoxia (HIF-1α signaling)
ISCU	miR-210-3p	Mitochondrial metabolism; Hipoxia (HIF-1α signaling)
FOXO1	miR-96-5p miR-183-5p	Proliferation/apoptosis escape
ZFP36L1	miR-96-5p miR-183-5p	Proliferation/apoptosis escape; Migration/invasion
INSIG1	miR-183-5p miR-210-3p	Proliferation/apoptosis escape; Migration/invasion; Lipogenesis

**Fig. 3:**
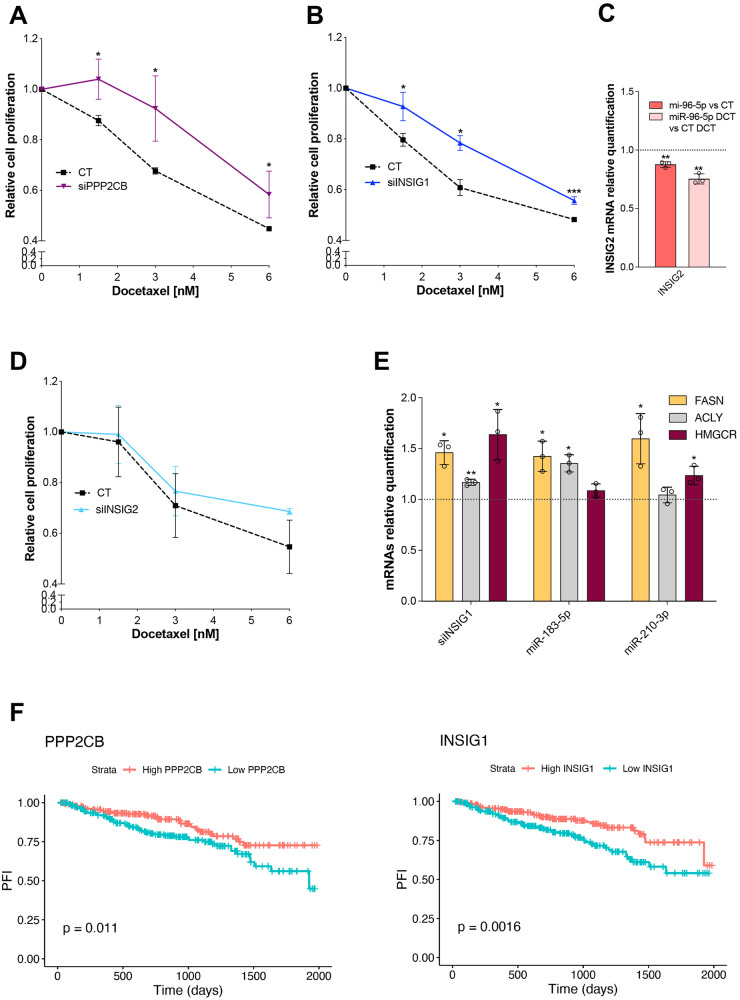
sDCT^R^-miRNAs modify DCT response of PCa cells by regulating PPP2CB and INSIG1. Cell proliferation after PPP2CB (**A**), INSIG1 (**B**), and INSIG2 (**D**) silencing in treated (with increasing DCT doses) compared to untreated samples. **C** Relative INSIG2 mRNA quantification by qRT-PCR after miR-96-5p overexpression in treated (3 nM) compared to untreated samples. **E** FASN, ACLY, and HMGCR mRNAs relative quantification by qRT-PCR after INSIG1 silencing or miR-183-5p or miR-210-3p overexpression in treated (3 nM DCT) compared to untreated samples. **F** Kaplan–Meier curves showing progression-free interval (PFI) relative to patients stratified by PPP2CB and INSIG1 expression level (high/low according to 0.5 quantile of log2(x + 1)). Log-rank test’s p-value is shown. Data are reported as mean ± SD of at least three independent experiments, *P < 0.05, **P < 0.01, ***P < 0.001 unpaired *t*-test (**A**–**E**).

We then investigated whether these targets were modulated by sDCT^R^-miRNAs in the PCa context. We overexpressed the three miRNAs in the DU-145 cells and quantified the target transcripts in the presence or absence of DCT treatment (Fig. 2B). Among the selected targets, 11 out of 20 were downregulated in PCa cells, in a few cases only after DCT treatment. It is worth noting that FOXO1, IGFBP3, and PDCD4 have been shown to play a role in DCT resistance in PCa [23–25]. We focused on PPP2CB and INSIG1. To investigate whether altering the expression of these targets could affect the DCT toxicity in PCa cells, we silenced PPP2CB and INSIG1 (Fig. S1C) and found that the sensitivity of DU-145 to DCT decreased (Fig. 3A, B). These results suggest that PPP2CB and INSIG1 act as mediators of miR-183-5p and miR-183-5p/miR-210-3p respectively.

INSIG1 is a target of both miR-183-5p and miR-210-3p but the validation type is “weak” according to miRTarBase 8.0. By using different predictive algorithms (miRWalk, miRDB, and TargetScan) we found a putative miR-183-5p binding site (identified by miRDB and TargetScan) and no miR-210-3p binding site in the INSIG1 3’ UTR. Nevertheless, Jo et al. [26] demonstrated that miR-183-5p does not affect INSIG1 expression by binding the putative INSIG1 3’UTR binding site. However, the same authors showed that miR-96-5p directly regulates INSIG2. We evaluated INSIG2 expression in miR-96-5p overexpressing cells treated with or without DCT and found that this miRNA downregulated INSIG2 in both situations (Fig. 3C). We then investigated whether alterations of INSIG2 expression affected DCT toxicity of PCa cells and found that INSIG2 silencing (Fig. S1C) did not significantly alter DCT sensitivity of DU-145 cells (Fig. 3D), suggesting that INSIG2 is not a mediator of miR-96-5p activity. Therefore, miR-183-5p and miR-210-3p affect PCa cell sensitivity to DCT by (possibly indirectly) decreasing INSIG1 levels.

The decrease of INSIG1 protein might contribute to the activation of the sterol regulatory element-binding protein (SREBP) pathway. We checked the expression of some SREBP targets (FASN, ACLY, and HMGCR) and found that overexpression of miR-183-5p or miR-210-3p as well as silencing of INSIG1 caused an increase of SREBP targets upon DCT treatment (Fig. 3E). Overall, our data suggest that miR-183-5p and miR-210-3p affect the DCT sensitivity of PCa cells by regulating INSIG1 expression and function.

Finally, similarly to sDCT^R^-miRNAs, we performed Kaplan-Meier analyses using PRAD TCGA dataset and found that patients with lower INSIG1 and PPP2CB expression had a worst PFI (Fig. 3F) and DFI (Fig. S2A, B). The same results were obtained for FOXO1 and PDCD4 (Fig. S2C–F). These data suggest that impairment of the miR-183-5p/miR-210-3p/INSIG1, miR-183-5p/PPP2CB, miR-183-5p/miR-96-5p/FOXO1 and miR-183-5p/PDCD4 regulatory axes are significantly associated with disease recurrence, suggesting a possible clinical relevance of the sDCT^R^-miRNAs in DCT resistance.

### Secreted sDCT^R^-miRNAs reduce DCT sensitivity in PCa cells

**Fig. 4:**
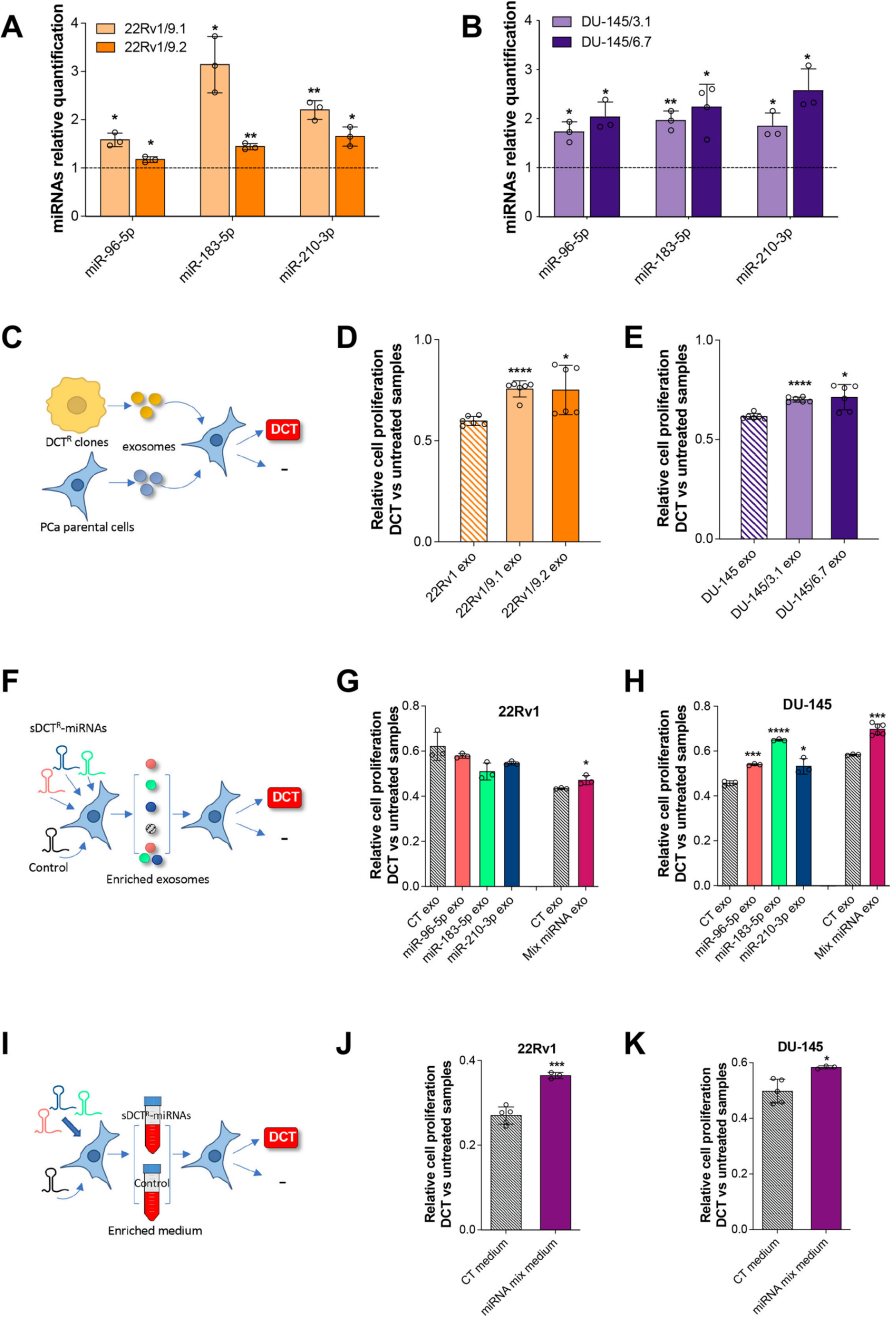
Secreted sDCT^R^-miRNAs exposure decreases the DCT sensitivity of PCa cells. Relative sDCT^R^-miRNAs quantification by qRT-PCR in the exosomes secreted by 22Rv1/9.1 and /9.2 DCT^R^ clones (**A**) and DU-145/3.1 and /6.7 DCT^R^ clones (**B**) compared to the exosome secreted by the correspondent parental cells. Relative cell proliferation of 22Rv1 (**D**) or DU-145 (**E**) cells exposed to 22Rv1/9.1, /9.2 or DU-145/3.1, /6.7 exosomes respectively in treated (3 nM DCT) compared to untreated samples. Relative cell proliferation of 22Rv1 (**G**) or DU-145 (**H**) cells after exposure of sDCT^R^-miRNAs enriched exosomes in treated (3 nM DCT) compared to untreated samples. PCa cells were exposed to exosomes enriched with a single sDCT^R^-miRNA or a mixture of exosomes enriched with a single sDCT^R^-miRNA (mix miRNAs exo). Relative cell proliferation of 22Rv1 (**J**) or DU-145 (**K**) cells exposed to conditionate medium recovered after the overexpression of an equimolar mix of the three sDCT^R^-miRNAs (miRNAs mix medium) in treated (3 nM DCT) compared to untreated samples. **C**, **F**, **I** Schematic representation of the exosomes/medium exposure experiments. Data are reported as mean ± SD of at least three independent experiments, *P < 0.05, **P < 0.01, ***P < 0.001 unpaired *t*-test.

Since sDCT^R^-miRNAs are specifically released from DCT^R^ PCa clones, we wondered whether these miRNAs could function as molecular messengers in cell-to-cell communication. More specifically, we asked whether sDCT^R^-miRNAs released from DCT^R^ PCa clones could alter the DCT response of PCa cells still sensitive to the drug. Therefore, we isolated the exosomes released from two 22Rv1 DCT^R^ clones (clone 22Rv1/9.1 and 22Rv1/9.2) and two DU-145 DCT^R^ clones (DU-145/3.1 and DU-145/6.7) and treated DU-145 or 22Rv1 cells sensitive to DCT with these exosomes. Before that, we made sure that: (i) we had successfully isolated vesicles ranging from 50 nm to 200 nm in diameter by using Nanosight tracking analysis (NTA) (Fig. S3); (ii) the sDCT^R^-miRNAs were not only more prominently released in the medium but also enriched in the exosomes (Fig. 4A, B). The results showed that treatment with exosomes from DCT^R^ clones (compared to 22Rv1 or DU-145 exosomes) decreased the DCT sensitivity of both 22Rv1 and DU-145 parental cells, suggesting that DCT^R^ clones may alter the behavior of PCa cells through signals from their exosomes (Fig. 4C–E).

To understand whether these signals were at least partially composed of sDCT^R^-miRNAs, we separately overexpressed the three miRNAs in both DU-145 and 22Rv1 cells and isolated the exosomes from the culture medium at 48 h post-transfection, when the miRNAs release was greatest (Fig. S4A–D). We then exposed both DU-145 and 22Rv1 cells to sDCT^R^-miRNA enriched exosomes and observed that the exposure decreased sensitivity to DCT albeit only in DU-145 cells (Fig. 4F–H). Next, we exposed both PCa cell lines to a mixture of exosomes loaded with each individual sDCT^R^-miRNA, and we observed that the DCT sensitivity decreased in both cell lines (Fig. 4F–H). Similarly, we exposed both PCa cell lines to the culture medium of cells overexpressing an equimolar mixture of the sDCT^R^-miRNAs and we observed decreased DCT sensitivity (Fig. 4I–K). Overall, our data show that exosomes or culture medium enriched with sDCT^R^-miRNAs decrease the sensitivity of PCa cells to DCT, suggesting that these miRNAs may function as molecular messengers in cell-to-cell communication. Moreover, the effect of the sDCT^R^-miRNA mixture was more effective than a single sDCT^R^-miRNA, suggesting that they may act synergistically.

### DCT^R^-miRNAs were associated with DCT response in PCa patients under DCT treatment

**Fig. 5:**
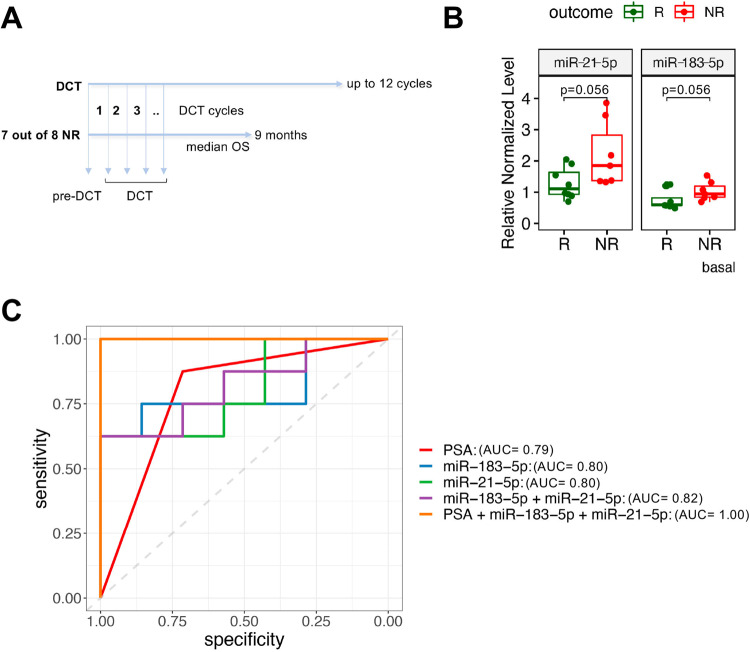
Some DCT^R^-miRNAs are associated with DCT resistance in PCa patients. **A** Schematic representation of samples collection. Overall survival was defined as the time from the first cycle of DCT to the time of death and calculated for 7 out of 8 NR pts. **B** Relative normalized qRT-PCR levels of DCT-miRNAs in serum of NR and R pts before DCT treatment. *p*-value was calculated using Wilcoxon rank sum test. **C** ROC curves of the classification models are shown for the sDCT^R^-miRNAs-based (singularly, in combination, and with PSA) and PSA-based regression analysis together with their AUC values.

Considering that DCT^R^-miRNAs are specifically released by PCa cells resistant to DCT, we investigated whether they could be detected in the blood of PCa patients and whether their levels could be related to the occurrence of DCT resistance. As an exploratory analysis, we recruited 16 metastatic PCa patients regardless of their hormone sensitivity status (metastatic castration-resistant (mCRPC) or metastatic hormone-sensitive (mHSPC) prostate cancer) (Table 3). Serum samples were collected before the first DCT treatment and during the treatment when patients went back to the hospital for DCT therapy (one treatment every two or three weeks) (Fig. 5A). At the first evaluation (after 4 cycles of DCT) patients were classified as “responders” (Rs) or “non-responders” (NRs) based on the following criteria (according to the Prostate Cancer Working Group 2 -PCWG2-): (i) increase in metastatic lesions; (ii) increase in prostate-specific antigen (PSA) level; (iii) clinical deterioration. Patients with 2 out of 3 criteria were considered NRs.

**Table 3:** Characteristics of patient’s cohort.

		Numbers of pts (total pts)	Median (range)
Responders		8 (16)	
Non responders		8 (16)	
mHSPC		1 (16)	
mCRPC		15 (16)	
Age (year)		16 (16)	75 (56–84)
Status	alive	9 (16)	
	dead	7* (16)	
PSA (μg/l)	before DCT	16 (16)	29.075 (0.33–693)
	after DCT	15 (16)	27.27 (0.21–832)
Gleason at diagnosis	<7	0 (16)	
	=7	4 (16)	
	>7	12 (16)	
Metastasis	bone only	4 (16)	
	bone, visceral	2 (16)	
	bone, nodal	6 (16)	
	bone, nodal, visceral	4 (16)	
Subsequent treatments	abiraterone	1 (16)	
	cabazitaxel	1 (16)	
	enzalutamide	2 (16)	
	radiotherapy	1 (16)	
	none	7 (16)	
	unknown	3 (16)	

^*^Patients were all non-responders.

We measured the level of DCT^R^-miRNAs (Table 1) in the serum samples collected before the first DCT treatment (basal) (15 out of 16 pts) and during DCT treatment (cycle 2) (10 out of 16 patients). Considering the DCT^R^-miRNAs levels at baseline, we found that miR-21-5p and miR-183-5p were higher (*p* < 0.06) in NRs versus Rs patients (Fig. 5B). Of note miR-10a-5p and miR-96-5p also showed higher level in NRs vs Rs patients but with p > 0.06, (Fig. S4E). Interestingly, unlike miR-21-5p, the level of miR-183-5p tended to remain higher in NRs vs Rs also at cycle 2, albeit with *p* > 0.06 (Fig. S4F).

Finally, we assessed the role of miR-183-5p and miR-21-5p as potential predictive biomarkers of response to DCT in our cohort. Using multivariate logistic regression analysis, we evaluated the association between baseline miRNA levels and response to therapy. Both individual miRNA and their combination were modeled. As shown by AUROC in Fig. 5C, the classification models based on miRNA levels are comparable to those based on PSA amount. Notably, the combination of the two-miRNAs (AUC = 0.82) performed slightly better than PSA (AUC = 0.79), and, in particular, the combination of the two-miRNAs with PSA greatly improved the predictive power of the classification model in discriminating between Rs and NRs (AUC = 1.00).

## Discussions

Although many efforts have been made to comprehend the mechanism of DCT resistance in PCa, the field has not advanced much in recent years. To gain more insight into this field, in a previous work we isolated PCa resistant clones from two PCa cell lines with different genetic backgrounds and identified the miRNAs specifically released by these clones (DCT^R^-miRNAs) [21]. In this work, we decided to re-analyze the NGS data and we identified additional DCT^R^-miRNAs (Table 1). In the list of DCT^R^-miRNAs shown in Table 1 three miRNAs previously identified in 22Rv1 DCT^R^ clones (i.e., miR-4792, miR-4532, and miR-5096) were excluded as they are considered “dead miRNA entry” in the new miRBase release 22.1 (2019).

Considering the identified DCT^R^-miRNAs, we realized that most of them could potentially affect DCT response of PCa cells by regulating pathways that might contribute to docetaxel resistance. Indeed, miR-96-5p induces several cellular survival pathways [27–29] or promotes apoptosis inhibition [30], EMT, invasion, and migration in PCa [31, 32]. Similarly, miR-210-3p exhibits oncogenic activity in PCa by promoting EMT, invasion, and migration [33, 34]. Of note, both miR-21-5p and miR-183-5p have been shown to impair DCT-induced apoptosis or DCT-induced reduction of cell viability by targeting PDCD4 and SPRY2, respectively, in PCa cells [25, 35]. We focused on miR-210-3p and the two members of the miR-183 cluster (miR-96-5p and miR-183-5p) (sDCT^R^-miRNAs). Interestingly, both miR-210-3p and miR-183 cluster are directly activated by HIF1α [36, 37] and thus are mediators of the hypoxic response. Activation of HIF1α signaling, which is usually triggered in tumor cells under hypoxic conditions, increases tumor cell survival [38, 39] and may contribute to taxane resistance [8, 40, 41]. By overexpressing sDCT^R^-miRNAs we demonstrated their ability to reduce sensitivity of PCa cells to DCT treatment.

Since a single miRNA can regulate hundreds of targets, we explored the molecular mediators through which they counteract DCT activity. We decided to narrow the field to direct sDCT^R^-miRNA targets using the validated target database (miRTarBase). As miRNAs are inhibitors of gene expression, we focused on those targets that are downregulated in PCa cells resistant to DCT and that play a role consistent with known mechanisms of DCT resistance (Table 2). We found that sDCT^R^-miRNAs regulate most of the selected targets in PCa cells at the mRNA level. We did not consider targets regulated by sDCT^R^-miRNAs through translation inhibition. However, translation inhibition is considered a lesser adopted miRNA regulatory mechanism as compared to target destabilization and consequent decay in post-embryonic cells [42]. Among the sDCT^R^-miRNA regulated targets, three targets are of particular interest: FOXO1, IGFBP3, and PDCD4. FOXO1 silencing decreases DCT toxicity in PCa cells by counteracting the DCT-dependent increases in nuclear FOXO1 and the resulting FOXO1-dependent cell death induction [23]. Similarly, Igarashi et al. [24] demonstrated that overexpression of IGFBP3 increases DCT toxicity in PCa cells, possibly impairing the IGF1R/IRS1/AKT pathway activation and, consequently, leading to apoptosis [43]. Finally, as previously mentioned, inhibition of PDCD4 by miR-21-5p decreased the efficacy of DCT treatment in PCa cells [25]. According to these data FOXO1, PDCD4, and IGFBP3 could be considered as mediators of DCT toxicity reduction driven by miR-183-5p/miR-96-5p, miR-183-5p, and miR-210-3p respectively.

We focused on PPP2CB and INSIG1 and demonstrated for the first time that silencing these two genes contributes to reducing DCT cytotoxicity in PCa cells. PPP2CB is one of the two isoforms of the catalytic subunit of serine/threonine-protein phosphatase 2 A (PP2A) [44]. In general, PP2A is considered a tumor suppressor and its dysregulation affects several physiological processes due to its involvement in most cellular pathways [44]. In particular, PP2A silencing in PCa cells has been shown to increase the expression of XIAP protein (via the PP2A/p-eIF4B/XIAP axis), which exerts its antiapoptotic effect by enhancing resistance to DCT [45]. Intriguingly, miR-1246, by directly targeting PPP2CB, impairs the function of the PP2A protein complex and leads to increased NF-kβ activity in mesenchymal stem/stroma cells [46]. Since NF-kβ activity has been associated with increased resistance of PCa cells to DCT [47, 48], we might speculate that miR-183-5p may counteract DCT cytotoxicity by inhibiting PP2A activity (through PPP2C2B inhibition) and, in turn, activating NF-kβ signaling. Interestingly, miR-210-3p sustains the activation of NF-kβ signaling in PCa cells by targeting negative regulators of the signaling [33].

INSIG proteins (INSIG1 and INSIG2) are important regulators of SREBP activity [49]. Indeed INSIGs, by binding the SCAP (SREBP cleavage-activating protein)/SREBPs complexes, retain SREBPs in the endoplasmic reticulum preventing SCAP/SREBPs translocation to the Golgi apparatus and hence SREBP pathway activation. Xu and colleagues [50] demonstrated that disrupting the interaction between INSIGs and SCAP (due to activation of the AKT/PCK1 axis in hepatocellular carcinoma cells) leads to activation of SREBPs and, consequently, lipogenesis that in turn increases cell proliferation. Therefore, a possible scenario entails that inhibition of INSIG1 expression by miR-183-5p and miR-210-3p increases DCT resistance by sustaining cancer cell survival and activating lipogenesis. This hypothesis is corroborated by the observation that either silencing of INSIG1 or overexpression of miR-183-5p and miR-210-3p increased the expression of lipid metabolism genes whose expression is directly regulated at the transcriptional level by SREBPs (FASN, ACLY, and HMGCR [50, 51]).

INSIG expression is regulated by miR-96-5p (INSIG2) (Fig. 3C) and miR-183-5p/miR-210-3p (INSIG1) in PCa (Fig. 2B). However, only INSIG1 silencing appears to significantly reduce DCT sensitivity. As previously mentioned, the validation of miR-183-5p/ and miR-210-3p/3’UTR INSIG1 interactions is classified as “weak” according to miRTarBase. Moreover, the miR-210-3p/3’UTR INSIG1 interaction is not predicted by any of the adopted algorithms. Conversely, although miR-183-5p/INSIG1 interaction is predicted by both TargetScan and miRDB, miR-183-5p does not appear to regulate INSIG1 through 3’UTR direct binding [26]. These data suggest that miR-183-5p and miR-210-3p regulate INSIG1 expression (and thus DCT sensitivity) in an indirect manner. However, the interaction miR-183-5p/INSIG1 was demonstrated by CLASH experiments [52] and, more specifically, it was shown that interaction occurs in the coding sequence (CDS) of INSIG1. Although miRNAs binding to the CDS is considered less effective [53, 54] some evidence confirm that miRNA-mediated regulation can occur on 5’UTR- or CDS-localized miRNA binding sites [55]. Therefore, direct regulation of INSIG1 by miR-183-5p through CDS interaction cannot be excluded.

Finally, according to Kaplan-Meier analyses, the expression of sDCT^R^-miRNAs and almost all the identified target mediators in PCa patients are directly (mRNAs)/inversely (miRNAs) associated with the time interval of tumor progression. These data suggest that the pathways regulated by these miRNAs play a pivotal role in disease recurrence and possibly in the appearance of DCT resistance in PCa patients.

Since sDCT^R^-miRNAs are specifically released by DCT resistant PCa cells we hypothesized they may function as messenger molecules in cell-to-cell communication. Firstly, we demonstrated that exposure of PCa cells to exosomes secreted by DCT^R^ clones, in which the level of sDCT^R^-miRNAs was higher than in parental cells exosomes, decreased DCT sensitivity of PCa cells. Secondly, we showed that also exposure to exosomes enriched in sDCT^R^-miRNAs reduced DCT cytotoxicity. These data suggest that the ability of exosomes of DCT^R^ clones to modify the DCT response of PCa cells is at least partially dependent on sDCT^R^-miRNAs. In support of this observation, some pieces of evidence have shown that sDCT^R^-miRNAs are involved in drug resistance by exerting a function in cell-to-cell communication. Exosomal miR-96-5p has been shown to increase cisplatin resistance in lung cancer cells possibly regulating LMO7 expression [56]. Two studies demonstrated that the horizontal transfer of exosomal miR-210-3p from gemcitabine-resistant to gemcitabine-sensitive pancreatic cancer cells [57] or from osimertinib-resistant to parental lung adenocarcinoma cells [58] antagonized gemcitabine induced apoptosis or increased osimertinib resistance, respectively. Finally, PCa derived exosomal miR-183-5p increased cell proliferation and migration/invasion by regulating TPM1 [59]. Overall, these observations support our findings that exosomal sDCT^R^-miRNAs that are secreted by DCT-resistant PCa cells are able to alter the drug response in DCT-sensitive PCa cells thereby limiting the DCT efficacy. This observation is relevant considering that DCT resistance, as well as cancer drug resistance in general, often arises within the TME in which crosstalk between heterogeneous cancer cells (contained within highly complex tumors) occurs.

We also evaluated whether DCT^R^-miRNAs (i) can be detected in the blood of PCa patients and whether they (ii) are associated to DCT resistance in patients undergoing DCT therapy. Although our cohort is limited, we found that high serum levels of miR-183-5p and miR-21-5p before DCT treatment are associated with a poorer response to DCT therapy. More importantly, we found that basal serum levels of these DCT^R^-miRNAs performed well in discriminating Rs and NRs patients, especially when used in combination with PSA. Interestingly, circulating miR-21-5p has already been identified as a potential predictive biomarker of DCT therapy efficacy in mCRPC patinets [60]. Also circulating miR-210-3p level has been shown to correlate with the response to DCT treatment in mCRPC patients [61]. In our cohort, although the miR-210-3p serum level did not change at baseline in NRs compared to Rs patients, it was higher in NRs versus Rs patients at cycle 2 although with *p* > 0.06 (data not shown). Therefore, identifying miRNAs specifically released by DCT resistant tumor cells in vitro seems to be a good strategy to identify potential predictive biomarkers of drug resistance. Considering these data, secreted miR-183-5p not only modulates DCT response in vitro but also correlates with the outcome of DCT therapy in patients with metastatic PCa.

In conclusion, we demonstrated that miR-96-5p, miR-183-5p, and miR-210-3p counteract DCT cytotoxicity in PCa cells by regulating FOXO1, IGFBP3, PDCD4, INSIG1, and PPP2CB likely by increasing survival pathways and escaping from DCT-induced apoptosis. Furthermore, we demonstrated that exosomal miR-96-5p, miR-183-5p, and miR-210-3p secreted by DCT-resistant PCa cells reduce DCT response in PCa cells still sensitive to the drug. Finally, in a small cohort of metastatic PCa patients, we showed that serum levels of miR-183-5p and miR-21-5p are associated with the occurrence of DCT resistance. Overall, our data indicate the importance of miRNA regulating cell-to-cell crosstalk in the mechanism of DCT resistance and highlight the potential clinical relevance of this phenomenon.

## Materials and methods

### Cells and culture conditions

DU-145 and 22Rv1 cell lines were grown in RPMI 1640 medium added of 10% FBS, 1% penicillin/streptomycin 2 mM, and 1% L-glutammine 2 mM (Euroclone). Cells were incubated at 37 °C in a humidified atmosphere containing 5% CO_2_.

### Transfection

Transient transfections of double-stranded miRNAs mimics (miR-96-5p, miR-183-5p, and miR-210-3p), and siRNAs (siPPP2CB, siINSIG1 and siINSIG2) or control (CT) (Supplementary Table S4) (Eurofins Genomics) in 22Rv1 and DU-145 cells were carried out using Lipofectamine 2000 (Thermo Fisher). 1 × 10^5^ cells (22Rv1) or 0.6 × 10^5^ cells (DU-145) per well were seeded in 12-wells dishes and 48 h after seeding cells were transfected with 60 nM miRNA mimic or siRNA using 2 µl of Lipofectamine according to the manufacturer’s protocol. After 6 h the medium was replaced and the cells were treated or not with 3 nM DCT (Taxotere, 20 mg/ml, Sanofi Aventis). After 48 h the cells were harvested and used for molecular and cellular assays.

### Cell proliferation

At specified time points, cells were fixed in 2% paraformaldehyde in PBS (Oxoid), and subsequently stained with 0.1% crystal violet (SIGMA) dissolved in 20% methanol (SIGMA) and let dry at room temperature. Cells were then lysed with 10% acetic acid and the optical density (OD 590 nm) of the solution, detected with ChroMate Microtetraplate Reader apparatus (Awareness Technology), was used to measure cell proliferation.

### Dose response curves

1 × 10^4^ cells (22Rv1) or 0.8 × 10^4^ cells (DU-145) were seeded per well in 24 wells dishes. After 48 h cells were transfected with 60 nM miRNAs or siRNAs and after 6 h the media were replaced and the cells treated or not with increasing DCT doses. After 48 h cells were fixed in 2% paraformaldehyde and processed for cell proliferation evaluation (previously described).

### Colony forming ability

Cells were transfected as previously described. 48 h after transfection cells were collected and seeded at a cell density of 800 (DU-145) or 1000 (22Rv1) cells/60 mm diameter culture dish to allow colony formation. After 24 h cells were treated or not with 3 nM DCT. After 10-12 days, dishes were stained with 0.1% CV dissolved in 20% methanol and the number of colonies counted.

### Total RNA isolation

Total RNA was extracted from 2 × 10^5^ to 5 × 10^5^ cells using the miRNeasy mini kit (Qiagen) following the manufacturer’s recommendations.

For serum/medium or exosome, RNA was extracted from 400 µl of medium/serum or 100 µl of PBS resuspended exosomes using the miRNeasy micro kit (Qiagen) with some modification. We used 5 volumes QIAzol Lysis Reagent as indicated in the miRNaesy serum/plasma kit (Qiagen) protocol. According to this protocol 5 µM cel-miR-39 (GenePharma) was added after the QIAzol step.

### Quantification of miRNAs and mRNAs (qRT-PCR)

1 µg of total cellular RNA was retrotranscribed using either the mir-X miRNA FirstStrand Synthesis kit (Takara) or the PrimeScript RT reagent kit with gDNA Eraser (Takara) for the miRNAs or the mRNAs quantification, respectively. For serum/plasma or exosome 4 µl RNA was retrotranscribed. The reverse transcription was made following the instructions given by the manufacturer. miRNAs and the mRNAs were quantified with Rotor-Gene Q 2plex (Qiagen), using the SsoAdvanced ™ SYBR ® Green Supermix (Bio-Rad), according to the protocol indicated by the manufacturer. The relative quantification was performed using the Rotor-Gene Q Software, normalizing to the internal controls (U6, SNORD55 and SNORD110 for intracellular miRNAs, cel-miR-39 for extracellular miRNAs, and GAPDH, ACTB, and HPRT for mRNAs). Primers were purchased from Eurofins Genomics and indicated in Table S5. All reactions were performed in triplicate and the results are the mean of three biological replicates.

### Exosome isolation, characterization and exposure

Exosomes were isolated from 48-h medium (or 48-h post transfection medium) using ultracentrifugation method as described in [62] with some modifications. After the 2 h centrifugation at 100,000xg, exosomes were suspended in PBS and centrifugated at 100,000xg for 1 h. At the end of the centrifugation steps exosomes were suspended in 100 µl PBS and filtered through a 0.2 µm filter.

To further confirm that the size of the obtained particles was consistent with exosomes, the Nanosight particle tracking analysis (NTA) was performed using the NanoSight LM10 (Malvern Panalytical) according to the manufacturer’s suggestions and with our previous studies [63–65] (Fig. S3).

Cells were exposed to 10^9^ exosomes/cm^2^, after 24 h treated or not with 3 nM DCT, and after 72 h proliferation was measured as previously described.

### Medium collection and exposure

The media were collected 48 h after transfection and 10x concentrated using Spin Concentrators 5 K MWCO (Agilent Technologies). Cells were treated with concentrated media and after 24 h treated or not with DCT. The proliferation was measured after 72 h as previously described.

### Clinical samples and correlation analysis

Patients with metastatic prostate cancer receiving docetaxel therapy (75 mg/mq every 3 weeks) were recruited from the Department of Medical Oncology, San Donato Hospital, Arezzo. Blood samples were collected before the first cycle of DCT therapy and prior to the DCT treatment during the following cycles in specific tube with clot activator for serum separation. The blood samples were first centrifugated at 1500xg for 15 min and then the serum phases were centrifugated at 12000xg for 10 min to completely remove blood cells and stored at −80 °C.

All involved subjects signed the written informed consent to treatment and related procedures. The study was approved by the local ethics committees (approved report n.282/CEAVSE 20th October 2015, outcome of the ethics committee n.364/STAFF 5th November 2015), and the clinical investigation was conducted according to the Declaration of Helsinki.

A binomial generalized linear model (GLM) was used to assess the association between identified miRNAs and serum prostate-specific antigen (PSA) levels with the response status of the patients in our cohort. The Area Under the Receiver Operating Characteristics curve (AUROC) was used to assess the classification models’ performances.

### NGS and differential miRNA expression analyses

For next generation sequencing (NGS) data analysis of DU-145 DCT^R^ clones and parental cells raw reads were analyzed as described in Vitiello et al. [66]. Briefly, raw sequences were filtered using FastQC software v.0.11.7 and trimmed using Cutadapt v.1.9.1 to remove adapter sequences. The remaining reads, with lengths between 17 bp and 35 bp were clustered for unique hits and mapped to pre-miRNA sequences of miRBase (rel. 21) using miRExpress software v 1.0.0. The differential analysis was performed using two different Bioconductor R packages (https://bioconductor.org/) such as edgeR (v.3.38.4) and DESeq2 (v.1.36.0) based on distinct statistical methods and widely used in differential analysis. Normalized and filtered data were variance stabilized by both DESeq2 and edgeR. We considered to be expressed those miRNAs with the sum of the reads of all samples greater than 20. In addition, their log2 values of fold change must be less than −0.4 or greater than 0.4 with adjusted *p*-values < 0.05. p-values were adjusted for multiple testing by Benjamini-Hochberg method (labeled FDR by edgeR and padj by DESeq2). To obtain the heatmap plot, we computed the euclidean distance between the normalized (according to DESeq2 method) mapped reads of DU-145 DCT^R^ clones and parental cells.

### Bioinformatic analysis

The identification of the direct targets/putative mediators of the DCT^R^-miRNAs activity was preformed using two different pipelines designed in R. Using the first pipeline, from the validated targets list miR-96-5p, miR-183-5p and miR-210-3p obtained from locally hosted miRTarbase 8.0 [67] (https://mirtarbase.cuhk.edu.cn/), we performed a Gene Enrichment Analysis (GEA) with the clusterProfiler 4.4 package [68, 69] and the following database: Gene Ontology (GO:BP, GO:CC e GO:MF) (http://geneontology.org/); Disease Ontology (DO) (https://disease-ontology.org/); KEGG database (https://www.genome.jp/kegg/); Network Cancer Gene (NCG) (http://ncg.kcl.ac.uk/): Reactome Pathway Database (https://reactome.org/); DisGeNet (https://www.disgenet.org/). We selected the DCT^R^-miRNAs direct targets significantly enriched pathways (*p*-adjust <0.05). Using the second pipeline, we selected downregulated transcripts in DCT-resistant PCa cells by exploiting a single-cell sequencing data published by Schnepp et al. [22]. In particular, we selected the transcripts downregulated in the single DCT^R^ cells derived from both PCa cell lines (significantly *q*-value < 0.05 in at least one line). By combining the genes identified by both pipelines we obtained the putative direct mediators of DCT^R^-miRNAs.

### Survival analysis on TGCA data

The Survival analysis was performed using both gene expression (mRNAs and miRNAs) and clinical data of patients (*n* = 495 for miRNAs and *n* = 499 for mRNAs) retrieved of PRAD dataset (Level 3 data) of the Cancer Genome Atlas (TCGA) retrieved from Xena Browser (https://xenabrowser.net/). Curated progression free interval (PFI) and disease-free interval (DFI) from the Pan-cancer Atlas paper [70] were used to build Kaplan–Meier curves which were compared between groups of patients with log-rank statistics using “survival v.3.2.7” and “surviminer 0.4.8” CRAN packages.

### Statistical Analyses

All experimental results are expressed as mean ± standard deviation (SD) of at least three independent experiments and data analyzed by two-tailed unpaired Student’s *t*-test (*P < 0.05, **P < 0.01, ***P < 0.001, ****P < 0.0001) and performed with GraphPad Prism. Detailed information regarding the statistical test and sample size applied for each experiment were visible in the figures and stated in the figure legends.

### Supplementary information

Supplementary Figures

Supplementary Tables

## Data Availability

All data generated or analyzed during this study are available from the corresponding authors upon reasonable request.
