# Supplementary material for: Secreted miR-210-3p, miR-183-5p and miR-96-5p reduce sensitivity to docetaxel in prostate cancer cells
Source: Cell Death Discov. 2023 Dec 8;9:445. doi: 10.1038/s41420-023-01696-4 (PMC10709610; doi:10.1038/s41420-023-01696-4)
Supplement: Supplementary file 1 — Supplementary Figures [file 41420_2023_1696_MOESM1_ESM.pdf]

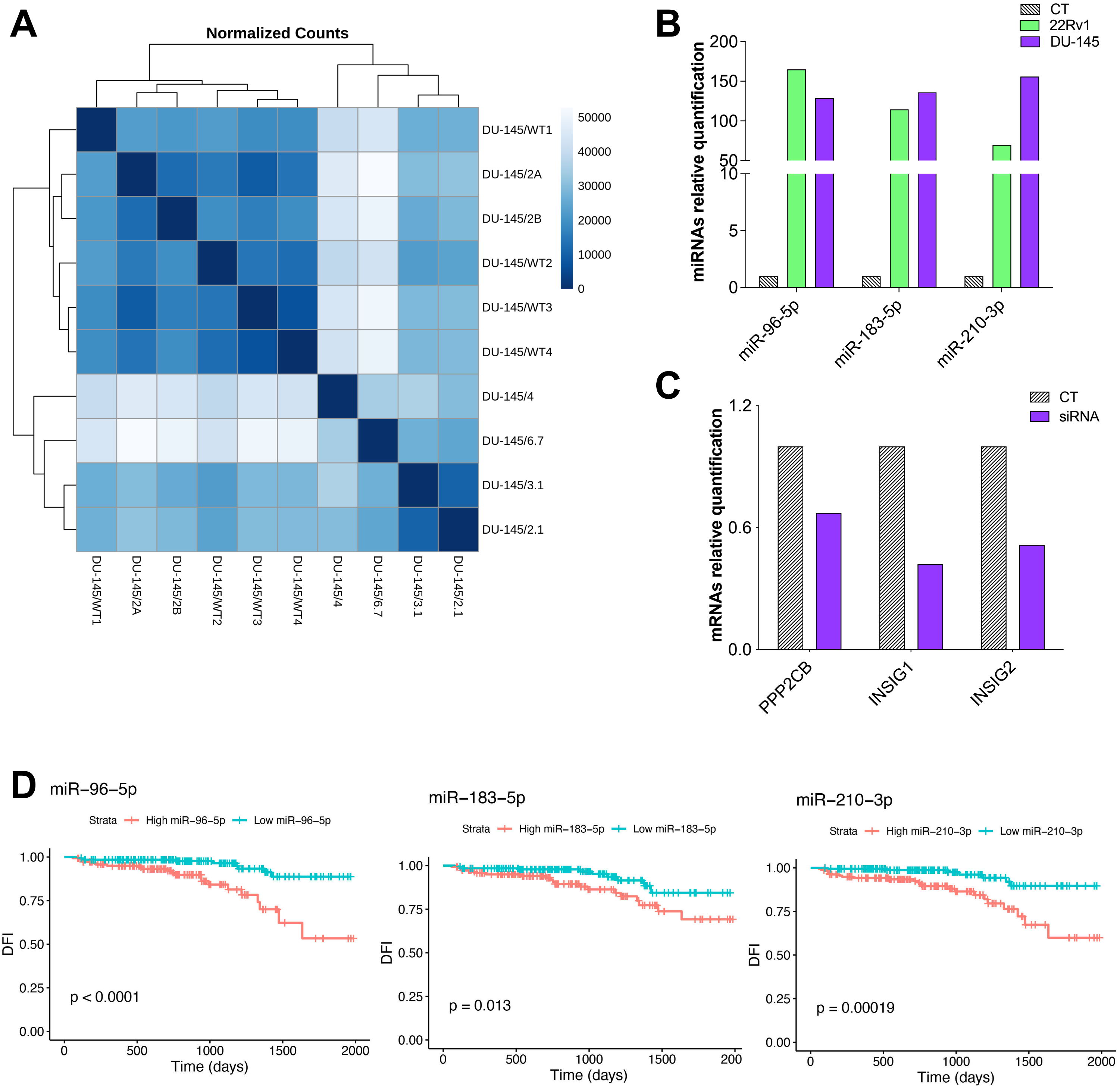

Supplementary Figure S1. **A** Hierarchical cluster obtained using the normalized read counts of DU-145 DCT^R clone and parental cells. **B** sDCT^R-miRNA relative quantification by qRT-PCR after sDCT^R-miRNAs overexpression in miRNAs compared to control transfected samples. **C** Relative quantification by qRT-PCR of sDCT^R-miRNA selected targets (PPP2CB, INSIG1 and INSIG2) after targets silencing in siRNAs compared to control transfected samples. **D** Kaplan-Meier curves showing disease free interval (DFI) relative to relative to patients stratified by sDCTR-miRNAs expression level (high/low according to 0.5 quantile of log2(total_RPM +1)). Log-rank test's p-value is shown.

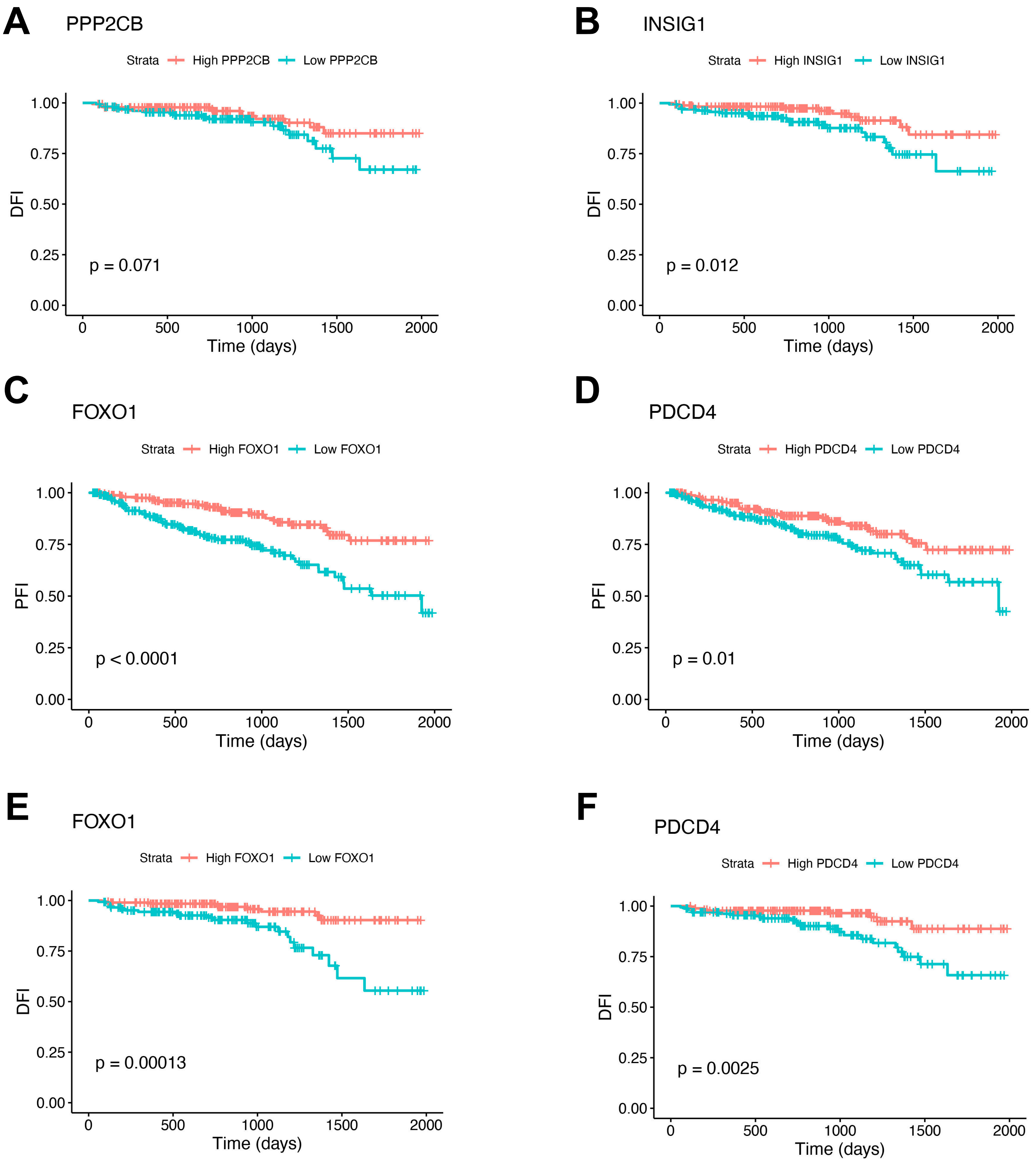

Supplementary Figure S2. Kaplan-Meier curves showing disease free interval (DFI) (**A-B, E-F**) and progression free interval (PFI) (**C-D**) relative to patients stratified by sDCT^{R}-miRNAs targets expression level (high/low according to 0.5 quantile of log2(x +1)). Log-rank test's p-value is shown.

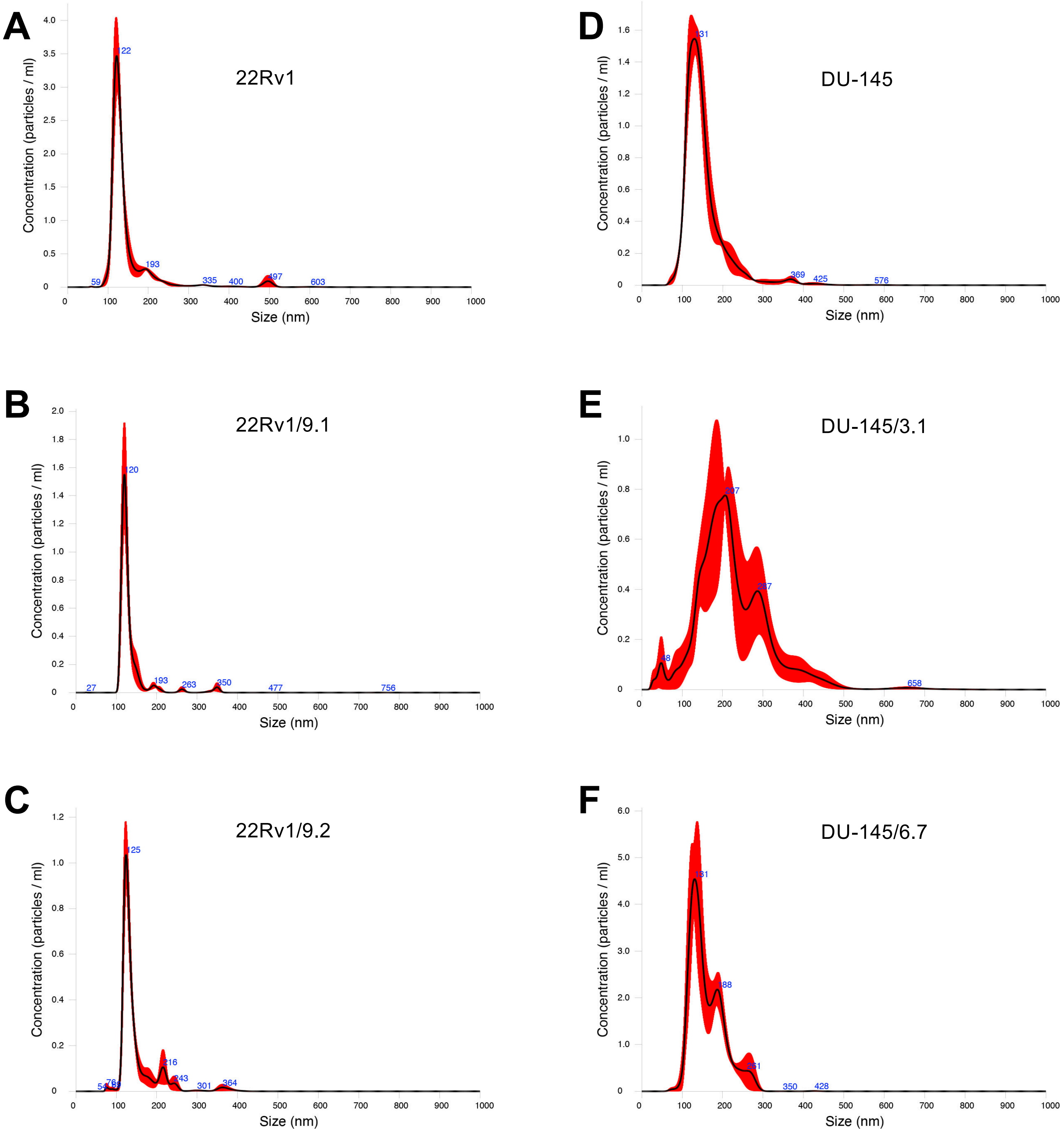

Supplementary Figure S3. Nanoparticle tracking analysis of DU-145 and 22Rv1 parental cells and DU-145/22Rv1-DCT^{R}-clones.

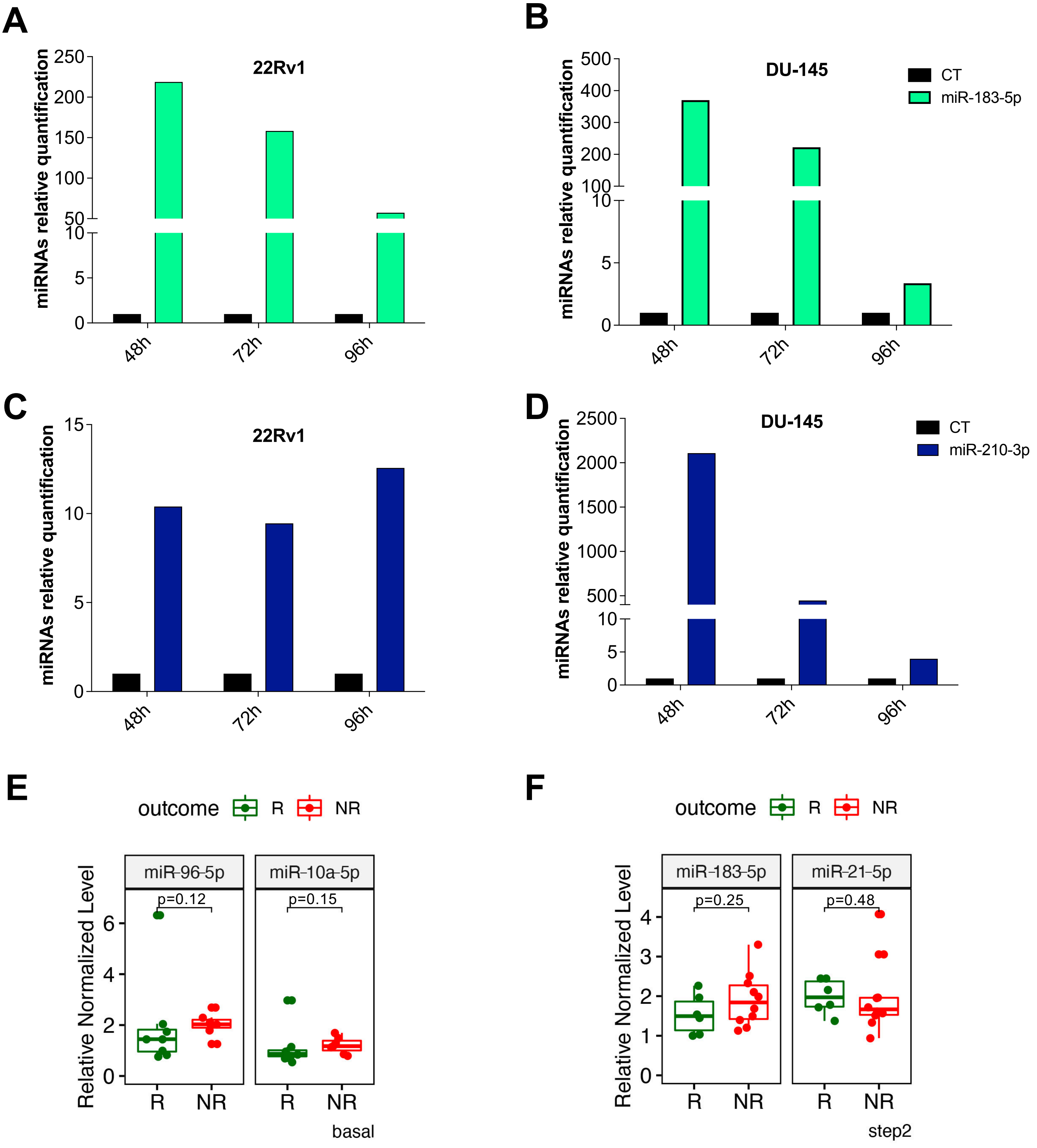

Supplementary Figure S4. Relative quantification of sDCT^{R}-miRNAs by qRT-PCR in the 22Rv1 (**A**, **C**) and DU-145 (**B**, **D**) growth media of cells transfected with miR183-5p (**A-B**) or miR-210-3p (**C-D**) compared to control transfected cells at different time points. Relative normalized qRT-PCR levels of DCT-miRNAs in serum of NR and R pts before (**E**) or at cycle 2 (**F**) of DCT treatment. p-value was calculated using the Wilcoxon rank sum test.
